# Characteristics and adaptability of iron- and sulfur-oxidizing microorganisms used for the recovery of metals from minerals and their concentrates

**DOI:** 10.1186/1475-2859-4-13

**Published:** 2005-05-06

**Authors:** Douglas E Rawlings

**Affiliations:** 1 Department of Microbiology, University of Stellenbosch, Private BagX1, Matieland, 7602, South Africa

## Abstract

Microorganisms are used in large-scale heap or tank aeration processes for the commercial extraction of a variety of metals from their ores or concentrates. These include copper, cobalt, gold and, in the past, uranium. The metal solubilization processes are considered to be largely chemical with the microorganisms providing the chemicals and the space (exopolysaccharide layer) where the mineral dissolution reactions occur. Temperatures at which these processes are carried out can vary from ambient to 80°C and the types of organisms present depends to a large extent on the process temperature used. Irrespective of the operation temperature, biomining microbes have several characteristics in common. One shared characteristic is their ability to produce the ferric iron and sulfuric acid required to degrade the mineral and facilitate metal recovery. Other characteristics are their ability to grow autotrophically, their acid-tolerance and their inherent metal resistance or ability to acquire metal resistance. Although the microorganisms that drive the process have the above properties in common, biomining microbes usually occur in consortia in which cross-feeding may occur such that a combination of microbes including some with heterotrophic tendencies may contribute to the efficiency of the process. The remarkable adaptability of these organisms is assisted by several of the processes being continuous-flow systems that enable the continual selection of microorganisms that are more efficient at mineral degradation. Adaptability is also assisted by the processes being open and non-sterile thereby permitting new organisms to enter. This openness allows for the possibility of new genes that improve cell fitness to be selected from the horizontal gene pool. Characteristics that biomining microorganisms have in common and examples of their remarkable adaptability are described.

## Review

### 1. Introduction

The solubilization of metals due to the action of microbes and the subsequent recovery of the metals from solution has deep historical roots that have been extensively reviewed [61,70]. Similarly, an indication of the number and sizes of the operations that employ microbes for the recovery of mainly copper, gold, cobalt and uranium has also been reviewed [61,72]. These processes use the action of microbes for one of two purposes. Either to convert insoluble metal sulfides (or oxides) to water soluble metal sulfates or as a pretreatment process to open up the structure of the mineral thereby permitting other chemicals to better penetrate the mineral and solubilize the desired metal. An example of the first type of process is the conversion of insoluble copper present in minerals such as covellite (CuS) or chalcocite (Cu_2_S) to soluble copper sulfate. An example of the second, is the removal of iron, arsenic and sulfur from gold-bearing arsenopyrite so that the gold that remains in the mineral is more easily extracted by subsequent treatment with cyanide. Both are oxidation processes, but where the metal to be recovered is extracted into solution the process is known as bioleaching, whereas when the metal remains in the mineral, bioleaching is an inappropriate term and the process should strictly be referred to as biooxidation. Nevertheless, the term bioleaching is frequently used for both.

Not all types of mineral are amenable to biologically-assisted leaching. In general, the mineral should contain iron or a reduced form of sulfur. Alternately, a mineral lacking in these compounds may be leached if it occurs together with another mineral that contains iron and reduced sulfur, provided that the mineral is subject to attack by ferric iron and/or sulfuric acid.

Metals in certain non-sulfide minerals may be solubilized by a process of complexation with oxalic, citric or other organic acids. These organic acids are typically produced by certain types of fungi and this type of metal solubilization process will not be discussed in this review [8].

This review will focus on properties that the various types of mineral biooxidation organisms have in common. However, before discussing these general characteristics it is necessary to describe briefly the mechanism of leaching and the technology of the metal recovery processes.

### 2. Mechanisms of bioleaching

Metal leaching is now recognized as being mainly a chemical process in which ferric iron and protons are responsible for carry out the leaching reactions. The role of the microorganisms is to generate the leaching chemicals and to create the space in which the leaching reactions take place. Microorganisms typically form an exopolysaccharide (EPS) layer when they adhere to the surface of a mineral [78] but not when growing as planktonic cells [22]. It is within this EPS layer rather than in the bulk solution that the biooxidation reactions take place most rapidly and efficiently and therefore the EPS serves as the reaction space [31,75,78,89].

The mineral dissolution reaction is not identical for all metal sulfides and the oxidation of different metal sulfides proceeds via different intermediates [80]. This has also been recently reviewed [75]. Briefly, a **thiosulfate mechanism **has been proposed for the oxidation of acid insoluble metal sulfides such as pyrite (FeS_2_) and molybdenite (MoS_2_), and a **polysulfide mechanism **for acid soluble metal sulfides such as sphalerite (ZnS), chalcopyrite (CuFeS_2_) or galena (PbS).

In the thiosulfate mechanism, solubilization is through ferric iron attack on the acid-insoluble metal sulfides with thiosufate being the main intermediate and sulfate the main end-product. Using pyrite as an example of a mineral, the reactions may be represented as:

FeS_2 _+ 6 Fe^3+ ^+ 3 H_2_O → S_2_O_3_^2- ^+ 7 Fe ^2+ ^+ 6 H^+ ^    (1)

S_2_O_3_^2- ^+ 8 Fe^3+ ^+ 5 H_2_O → 2 SO_4_^2- ^+ 8 Fe^2+ ^+ 10 H^+ ^   (2)

In the case of the polysulfide mechanism, solubilization of the acid-soluble metal sulfide is through a combined attack by ferric iron and protons, with elemental sulfur as the main intermediate. This elemental sulfur is relatively stable but may be oxidized to sulfate by sulfur-oxidizing microbes such as *Acidithiobacillus thiooxidans *or *Acidithiobacillus caldus *(reaction 5 below).

MS + Fe^3+ ^+ H^+ ^→ M^2+ ^+ 0.5 H_2_S_*n *_+ Fe ^2+ ^(n ≥ 2)     (3)

0.5 H_2_S_*n *_+ Fe^3+ ^→ 0.125 S_8 _+ Fe^2+ ^+ H^+ ^    (4)



The ferrous iron produced in reactions (1) to (4) may be reoxidized to ferric iron by iron-oxidizing microorganisms such as *Acidithiobacillus ferrooxidans *or bacteria of the genera *Leptospirillum *or *Sulfobacillus*.



The role of the microorganisms in the solubilization of metal sulfides is, therefore, to provide sulfuric acid (reaction 5) for a proton attack and to keep the iron in the oxidized ferric state (reaction 6) for an oxidative attack on the mineral.

### 3. Effect of temperature

Bioleaching processes are carried out at a range of temperatures from ambient to a demonstration plant that has been operated at 80°C [72]. As would be expected, the types of iron- and sulfur-oxidizing microbes present differ depending on the temperature range. The types of microbes found in processes that operate from ambient to 40°C tend to be similar irrespective of the mineral, as are those within the temperature ranges 45–55°C and 75–80°C. As described below, there are two broad categories of biologically-assisted mineral degrading processes. An ore or concentrate is either placed in a heap or dump where it is irrigated or a finely milled mineral suspension is placed in a stirred tank where it is vigorously aerated. In general, mineral solubilization processes are exothermic and when tanks are used, cooling is required to keep the processes that function at 40°C at their optimum temperature. At higher temperatures the chemistry of mineral solubilization is much faster and in the case of minerals such as chalcopyrite, temperatures of 75–80°C are required for copper extraction to take place at an economically viable rate.

### 4. Commercial metal extraction operations

#### 4.1. Heap leaching processes

Commercial bioleaching can take place using what may be considered to be a low technology process, the irrigation of waste ore dumps [13]. The metal recovery process may be made more efficient by the construction and irrigation of especially-designed heaps rather than by the irrigation of an existing dump that has not been designed to optimize the leaching process [13,72,81]. When building a heap, agglomerated ore is piled onto an impermeable base and supplied with an efficient leach liquor distribution and collection system. Acidic leaching solution is percolated through the crushed ore and microbes growing on the surface of the mineral in the heap produce the ferric iron and acid that result in mineral dissolution and metal solubilization. Aeration in such processes can be passive, with air being draw into the reactor as a result of the flow of liquid, or active with air blown into the heap through piping installed near the bottom. Metal-containing leach solutions that drain from the heap are collected and sent for metal recovery [81]. Heap reactors are cheaper to construct and operate and are therefore more suited to the treatment of lower grade ores. However, compared with tank reactors, heap reactors are more difficult to aerate efficiently and the undesirable formation of gradients of pH and nutrient levels as well as liquor channeling are difficult to manage. Furthermore, although one can rely on the natural movement of microbes to eventually inoculate the heap, initial rates of bioleaching can be improved by effective heap inoculation, but this is difficult to achieve.

Copper is the metal recovered in the largest quantity by means of heap reactors [reviewed in [61,72]]. Although comparisons are difficult as data are presented in different ways, examples of large copper leaching operations are those by Sociedad Contractual Minera El Abra and the Codelco Division Radimiro Tomic both in Chile and producing 225 000 and 180 000 tonnes Cu per annum respectively. Gold ore is also pretreated by bioleaching in heaps by Newmont Mining, in the Carlin Trend region, Nevada, USA.

#### 4.2. Tank leaching processes

In stirred tank processes highly aerated, continuous-flow reactors placed in series are used to treat the mineral. Finely milled mineral concentrate or ore is added to the first tank together with inorganic nutrients in the form of ammonia- and phosphate-containing fertilizers. The mineral suspension flows through series of highly-aerated tanks that are pH and temperature-controlled [23,70,93]. Mineral solubilization takes place in days in stirred-tank reactors compared with weeks or months in heap reactors. Stirred tank reactors that operate at 40°C and 50°C have proven to be highly robust and very little process adaptation is required for the treatment of different mineral types [68]. A major constraint on the operation of stirred tank reactors is the quantity of solids (pulp density) that can be maintained in suspension. This is limited to about 20% as at pulp densities >20%, physical mixing and microbial problems occur. The liquid becomes too thick for efficient gas transfer and the shear force induced by the impellers causes physical damage to the microbial cells. This limitation in solids concentration plus considerably higher capital and running costs in tank compared with heap reactors has meant that the use of stirred reactors has been restricted to high value minerals or mineral concentrates [72].

Stirred tanks are used as a pretreatment process for gold-containing arsenopyrite concentrates with the first of these having been built at the Fairview mine, Barberton, South Africa in 1986 [73,93]. The largest is at Sansu in the Ashanti goldfields of Ghana, West Africa. These two operations currently treat 55 and 960 tonnes of gold concentrate per day respectively. Another example is the use of stirred tanks to treat 240 tonnes of cobalt-containing pyrite in 1300 m^3 ^tanks at Kasese, Uganda [[14], reviewed in [72]].

#### Types of Microorganisms

In general, the types of microorganisms found in heap-leaching processes are similar to those found in stirred tank processes, however, the proportions of the microbes may vary depending on the mineral and the conditions under which the heaps or tanks are operated. In processes that operate from ambient temperatures to about 40°C, the most important microorganisms are considered to be a consortium of Gram-negative bacteria. These are the iron- and sulfur-oxidizing *Acidithiobacillus ferrooxidans *(previously *Thiobacillus ferrooxidans*), the sulfur-oxidizing *Acidithiobacillus thiooxidans *(previously *Thiobacillus thiooxidans*) and *Acidithiobacillus caldus *(previously *Thiobacillus caldus*), and the iron-oxidizing leptospirilli, *Leptospirillum ferrooxidans *and *Leptospirillum ferriphilum *[18,29,32,34,94]. If ferrous iron is added to the leaching solutions (lixiviants) that are circulated through a heap or dump, then *At. ferrooxidans *may dominate the iron-oxidizers. In continuous flow, stirred tank processes, the steady state ferric iron concentration is usually high and under such conditions *At. ferrooxidans *is less important than a combination of *Leptospirillum *and *At. thiooxidans *or *At. caldus *[71]. Gram-positive iron and sulfur-oxidizing bacteria related to *Sulfobacillus thermosulfidooxidans *have also been identified [29]. The consortium of bioleaching microbes frequently includes acidophilic heterotrophic organisms such as bacteria belonging to the genus *Acidiphilium *[38] or *Ferroplasma*-like archaea [33,95]. A fluidized-bed reactor operating at 37°C and pH 1.4 was dominated by *L. ferriphilum *with a small proportion of *Ferroplasma*-like archaea [47]. 'Heterotrophically inclined' microbes are believed to assist the growth of iron-oxidizing bacteria like *At. ferrooxidans *and the leptospirilli [36,43]. This is thought to be due to their ability to provide essential nutrients or to remove toxic organic compounds or other inhibitory substances. How much this ability contributes to the overall mineral biooxidation efficiency of a microbial consortium in practice is still unclear [45].

There are fewer commercial processes that operate in the 45–50°C range and therefore studies on microorganisms that dominate these bioleaching consortia have been less well reported. Rawlings et al., [71] identified *At. caldus *and a species of *Leptospirillum *as being the dominant microbes in a continuous-flow biooxidation tanks processing several mineral ores operating in this temperature range. *At. caldus*, *Sulfobacillus thermosulfidooxidans *and bacteria of the informally recognized species '*Sulfobacillus montserratensis*' together with an uncultured thermal soil bacterium were found to dominate the consortium of organisms oxidizing chalcopyrite concentrate at 45°C. The same bacteria dominated the culture irrespective of whether chalcopyrite, pyrite or an arsenic pyrite concentrate was being oxidized [26]. In a pilot scale, stirred-tank operation in which three tanks in series were used to treat a polymetallic sulfide ore at 45°C, *At. caldus*-like, *L. ferriphilum*-like and *Sulfobacillus*-like bacteria were found to dominate the first tank [59]. The proportions of these bacteria decreased in the second tank with the numbers of *At. caldus *and *Ferroplasma*-like archaea being equally dominant. The *Ferroplasma*-like archaea completely dominated the third tank with the number of leptospirilli being reduced to undetectable levels. When combinations of pure cultures were tested, a mixed culture containing both autotrophic (*Leptospirillum *MT6 and *At. caldus*) and heterotrophic moderate thermophiles (*Ferroplasma *MT17) was the most efficient [60]. The presence of *Ferroplasma*-like organisms is being increasing recognized in bioleaching processes that operate at very low pH (1.4 or less). These archaea appear to be able to oxidize minerals like pyrite in pure culture although not without a small quantity of yeast extract. Species of the gram-positive genus, *Acidimicrobium *[16] may occur together with sulfobacilli in cultures that grow at 45°C.

There are even fewer reports on types of microbes that occur in mineral treatment processes that operate at temperatures >70°C than at lower temperatures. However, it is clear that these biomining consortia are dominated by archaea rather than bacteria, with species of *Sulfolobus *and *Metallosphaera *being most prominent [54,57]. *Sulfolobus metalicus *has been found to dominate at 70°C but this archeaon is probably excluded at higher temperatures with other *Metalosphaera*-like and *Sulfolobus*-like archaea dominating at 80°C. Archaea belong to the genus *Acidianus *such as *Ad. ambivalensi *or *Ad. infernus *are also capable of growing at high temperature (90°C for *Ad. infernus*) on reduced sulfur and at low pH. However, the contribution of these organisms to industrial bioleaching is not well-established [54].

### 5. General characteristics of mineral degrading bacteria

As would be gathered from the above, the most important microbes involved in the biooxidation of minerals are those that are responsible for producing the ferric iron and sulfuric acid required for the bioleaching reactions. These are the iron- and sulfur-oxidizing chemolithrophic bacteria and archaea [70]. Irrespective of the type of process or temperature at which they are employed, these microbes have a number of features in common that make them especially suitable for their role in mineral solubilization. Four of the most important characteristics are; a) they grow autotrophically by fixing CO_2 _from the atmosphere; b) they obtain their energy by using either ferrous iron or reduced inorganic sulfur compounds (some use both) as an electron donor, and generally use oxygen as the electron acceptor; c) they are acidophiles and grow in low pH environments (pH.1.4 to 1.6 is typical) and d) they are remarkably tolerant to a wide range of metal ions [25], though there is considerable variation within and between species. Each of the these characteristics will be dealt with in the sections that follow.

The modest nutritional requirements of these organisms are provided by the aeration of an iron- and/or sulfur-containing mineral suspension in water or the irrigation of a heap. Small quantities of inorganic fertilizer can be added to ensure that nitrogen, phosphate, potassium and trace element limitation does not occur.

A further advantageous characteristic of mineral biooxidation operations is that they are usually not subject to contamination by unwanted microorganisms. In the case of continuous-flow tank leaching processes, the continual wash-out of mineral together with their attached microbes as well as the organisms in suspension provides strong selection for improved microorganisms.

### 6. Nutrition

#### 6.1 Autotrophy

Microorganisms that drive the mineral degradation processes are autotrophic and obtain their carbon for cell mass synthesis from the carbon dioxide in the air used to aerate the process. Heterotrophic microorganisms that live off waste products produced by the autotrophs are usually also present and there is some evidence that these heterotrophs might assist the process [45]. Mineral degradation processes differ from the vast majority of other commercial processes that employ microorganisms where an organic substrate is necessary to provide the carbon source and energy required for microbial growth. If it were necessary to feed the microorganisms required for mineral degradation with a carbon source (e.g. molasses), commercial mineral biooxidation processes would be unlikely to be viable.

Bacteria such as the acidothiobacilli and leptospirilli, fix CO_2 _by the Calvin reductive pentose phosphate cycle, using the enzyme ribulose 1,5-biphosphate carboxylase (RuBPCase or Rubisco) [92]. The CO_2 _concentration present in air is generally sufficient to avoid carbon limitation when bacteria such as *Acidithiobacillus ferrooxidans *are growing on ferrous iron. This bacterium probably responds to CO_2 _limitation by increasing the cellular concentration of RuBPCase [17]. *At. ferrooxidans *strain Fe1 has been reported to have two identical copies of the structural genes for RuBPCase (although the flanking regions are different, [49]) which are separated by more than 5 kb [48]. The reason for this duplication has not been tested.

*At. ferrooxidans *is considered to be an obligate autotroph but has been shown to use formic acid as a carbon source provided that it was grown in continuous culture and the formic acid was fed in sufficiently slowly for the concentration to remain low [65]. Similarly, genes for a formate hydrogenlyase complex have been located on the genome of *Leptospirillum *type II and it is therefore likely to also grow on formate [92]. However, like CO_2_, formic acid has a single carbon atom and when lysed by the cell formate may be assimilated by the Calvin cycle in much the same way as CO_2_. Whether the ability to use formate is of value in commercial processes is not clear.

In the case of several of the other bacteria, such as the moderately thermophilic *Sulfobacillus thermosulfidooxidans*, 1% v/v CO_2_-enriched air is required for rapid autotrophic growth in pure culture. This may be partly because the solubility of CO_2 _is reduced at 50°C and partly because these bacteria are known to be inefficient at CO_2 _uptake. *Sulfobacillu*s species are nutritionally versatile and also capable of heterotrophic growth [16,55].

Most members of the archaea are heterotrophic, although certain species of the genus *Sulfolobus *have been reported to grow autotrophically. Details of the CO_2_-fixation pathway are unknown although it has been suggested that acetyl-CoA carboxylation may be a key step and that the synthesis of biotin carboxylase and biotin-carboxyl-carrier protein are increased under conditions of CO_2 _limitation [54]. This complex is encoded by genes adjacent to genes encoding a putative propionyl-CoA carboxyl transferase and together these observations are in agreement with the suggestion that *Acidianus brierleyi *has a modified 3-hydroxypropionate pathway for CO_2 _fixation [41]. Other types of archaea such as the *Ferroplasma *have the genes necessary to fix carbon dioxide via the reductive acetyl CoA pathway [92]. Like *Sulfobacillus *spp., autotrophic growth of *Sulfolobus *spp. is enhanced in 1% CO_2_-enriched air [54].

#### 6.2 Nitrogen, phosphate and trace elements

Based on dry weight, nitrogen is the next most important element after carbon for the synthesis of new cell mass. Ammonium levels of 0.2 mM have been reported to be sufficient to satisfy the nitrogen requirement of *At. ferrooxidans *[91]. High concentrations of inorganic or organic nitrogen are inhibitory to iron oxidation. Exactly how much nitrogen needs to be present in a growth medium will be dependent on the quantity of cell growth to be supported. Ammonia is highly soluble in acid solutions and it has been found that traces of ammonia present in the air can be readily absorbed into growth media. Therefore determination of the exact nitrogen requirements is difficult to estimate. In commercial operations, inexpensive fertilizer grade ammonium sulfate is typically added to biooxidation tanks or bioleaching heaps to ensure that sufficient nitrogen is available [23].

The ability of *At. ferrooxidans *to reduce atmospheric dinitrogen to ammonia was reported and the genes for the enzyme nitrogenase (*nifHDK*) were cloned several years ago [52,64,69]. The ability to fix nitrogen is probably a general property of *At. ferrooxidans *as at least fifteen strains of *At. ferrooxidans *have been shown to contain the nitrogenase genes (Rawlings, unpublished). *L. ferrooxidans *was also shown to contain *nifHDK *genes, to reduce acetylene to ethylene (a common test for nitrogenase activity) and at the same time to oxidize ferrous to ferric iron at low oxygen concentrations [56]. This activity was repressed by ammonia, a strong indication of the nitrogen fixing activity. The nitrogen fixing (*nif*) operon and many of the *nif *regulatory elements of a *L. ferrooxidans *from the Tinto river have been isolated and sequenced [62,63]. Interestingly analysis of the genome of *Leptospirillum *type II (*L. ferriphilum*) indicated the absence of genes for nitrogen fixation in this species [92].

Nitrogenase enzyme activity is inhibited by oxygen. It was found that *At. ferrooxidans *growing on iron did not fix nitrogen when aerated, but began to fix nitrogen once the oxygen concentration had fallen [52]. Therefore, how much nitrogen fixation takes place in highly aerated biooxidation tanks or heaps is uncertain. However, the aeration of heaps is not homogenous and nitrogen fixation could take place in parts of a heap where the oxygen is absent or its concentration is sufficiently low. The sensitivity of nitrogenase to oxygen poses a special problem for leptospirilli because, as far as is known, it uses only iron as its electron donor and is probably obligately aerobic. One mechanism by which nitrogenase can be protected against oxygen is respiratory protection, whereby rapid consumption of oxygen by a cytochrome oxidase is maintains a low oxygen concentration compatible with nitrogen fixation. It has been suggested that cytochrome *bd *is responsible for respiratory protection in *At. ferrooxidans *[10]. It has been found that *Leptospirillum *type II also has genes encoding both *ccb3 *and *bd *terminal oxidases even though it has no nitrogenase [92]. One can speculate that if cytochrome *bd *is also present in *L. ferrooxdans*, this cytochrome could be responsible for respiratory protection of its nitrogenase.

### 7. Energy sources

As described in a previous section, the solubilization of minerals is considered to be a chemical process that results from the action of ferric iron and/or acid, typically sulfuric acid. Therefore, irrespective of the temperatures at which they grow at, the microorganisms that play the major role in the leaching of metals from minerals are either iron- or sulfur-oxidizing organisms. The iron and sulfur serve as electron donors during respiration.

#### 7.1 Iron oxidation

Ferrous iron is readily oxidized to ferric iron and in this way it can serve as an electron donor. The Fe^2+^/Fe^3+ ^redox couple has a very positive standard electrode potential (+770 mV at pH 2). As a result only oxygen is able to act as a natural electron acceptor and in the presence of protons with the product of the reaction being water (O_2_/H_2_O +820 mV at pH 7). The use of iron as an electron donor will therefore occur only during aerobic respiration. However, under aerobic conditions, ferrous iron spontaneously oxidizes to ferric iron unless the pH is low. Therefore, extremely acidophilic bacteria are able to use ferrous iron as an electron donor in a manner that is not possible for bacteria that grow at neutral pH. Because the difference in redox potential between the Fe^2+^/Fe^3+ ^and O_2_/H_2_O redox couples is small and because only one mole of electrons is released per mole of iron oxidized, vast amounts of ferrous iron need to be oxidized to produce relatively little cell mass. These large quantities of iron are not transported through cell membrane but remain outside of the cell and each ferrous iron atom simply delivers its electron to a carrier situated in the cell envelope (see below).

**Figure 1:**
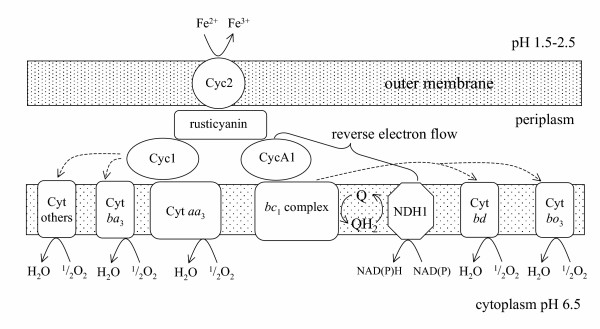
Model of the iron oxidation electron transport pathway of *At. ferrooxidans *based partly on references [10, 75]. Electrons are transferred from the membrane-located cytochrome *c *2 [97] to rusticyanin and then along one of two paths. The downhill path is via cytochrome *c*_4 _(Cyt1) to cytochrome *aa*_3 _[2] or the uphill, reverse electron transport path via cytochrome *c*_4 _(CytA1) to a *bc*_1 _I complex and a NADH-Q oxidoreductase [28]. *At. ferrooxidans *has up to twelve cytochromes *c *[98] and a variety of cytochrome oxidases some of which appear to play different roles depending on whether iron or sulfur is being oxidized [10]. The NADH is responsible for mercury reduction using a MerA mercuric reductase and the cytochrome *aa*_3 _is required to reduce mercury via the unique iron dependent mechanism discovered in *At. ferrooxidans *[84].

The mechanism of iron oxidation has been most extensively studied for the bacterium *At. ferrooxidans*. A model for iron oxidation is shown in Figure 1. This bacterium contains a *rus *operon that is proposed to encode for the electron transport chain that is used during the oxidation of ferrous iron [2]. This operon consists of genes for an *aa*_3_-type cytochrome oxidase, a high molecular weight outer membrane located cytochrome-*c *(Cyc2) [97], a *c*_4_-type cytochrome, a low molecular weight copper-containing protein rusticyanin (from which the operon derives its name) and an ORF proposed to encode a periplasmic protein of unknown function. The detection of rusticyanin has been linked to the growth of *At. ferrooxidans *on iron and it has been shown that the expression of the *rus *operon was 5- to 25-fold higher during growth on iron compared with sulfur [99]. Indeed, it has been calculated that up to 5% of the total cell protein of *At. ferrooxidans *when grown on iron consists of rusticyanin [19]. It has been suggested that rusticyanin probably functions as an electron reservoir in such a way that it readily takes up electrons available at the outer membrane and channels them down the respiratory pathway [76]. Rusticyanin serves as redox buffering function ensuring that the outer membrane Cyc2 electron acceptor remains in a fully oxidized state, ready to receive electrons from ferrous iron even in the presence of short-term fluctuations of oxygen. Interestingly aporusticyanin has been implicated in the adhesion of *At. ferrooxidans *cells to pyrite [4]. Although the *rus *operon is clearly involved in iron oxidation, it is not yet known whether the components of the operon are sufficient for iron the electron transport system or whether other components such as the *iro *gene for a high redox potential iron oxidase (HiPIP) might also play a role [50]. HiPIPs might not be present in all strains of *At. ferrooxidans *and might play a bigger role in sulfur oxidation than iron oxidation.

A question that has intrigued researchers is whether the iron-oxidation electron transport chains of different organisms are related. Bob Blake (Xavier University) and colleagues have investigated components of iron oxidation in at least five different acidophilic microorganisms, three bacteria (*Acidithiobacillus ferrooxidans*, unidentified bacterium m1, *Leptospirillum ferrooxidans*), and two archaea (*Sulfobacillus metallicus *and *Metallosphaera sedula*) [5,6]. In all five organisms the components of the electron transport chain were very different and the conclusion was that the ability to use ferrous iron as an electron donor has probably evolved independently at several times.

Although iron oxidation is best studied in *At. ferrooxidans*, enough is known to suggest that the mechanism in *L. ferrooxidans *(and presumably *L. ferriphilum*) must be substantially different. Whereas, *At. ferrooxidans *was capable of growth on ferrous iron at redox potentials of up to about +800 mV, *L. ferrooxidans *was capable of oxidation at redox potentials of closer to +950 mV [7,37]. The effect of this is that although *At. ferrooxidans *can outgrow *L. ferrooxidans *at high ratios of ferrous to ferric iron (as happens during the earlier stages of iron oxidation), *L. ferrooxidans *outcompetes *At. ferrooxidans *once the ferric iron concentration becomes high [74]. In a microbial community genome sequencing project, Banfield and co workers [92] reported the assembly of an almost complete genome of *Leptospirillum *group II, thought to be the same as *L. ferriphilum*. This genome contained a red cytochrome, presumably the same as the red cytochrome previously identified in *L. ferrooxidans *[5]. Other components typical of electron transport chains included putative cytochrome *cbb*_3_-type haeme-copper terminal oxidases and cytochrome *bd*-type quinol oxidases. A putative electron transport chain for *Leptospirillum *group II was constructed for both downhill respiration and uphill NADH synthesis electron flows.

#### 7.2 Sulfur as an energy source

The acid responsible for the very low pH environment in which extreme acidophiles are found is most often sulfuric acid. This sulfuric acid is produced by the oxidation of RISCs (reduced inorganic sulfur compounds). For biological oxidation to occur, the RISCs serve as an electron donor with oxygen serving as the energetically most favourable electron acceptor. The potential amount of energy that can be made available when a sulfur atom from a sulfide ore is oxidised to sulfate is much greater than when iron is oxidized [66]. Naturally occurring RISCs are present wherever sulfide-containing minerals are exposed to the surface. A variety of RISCs are released as a result of the chemical reaction of sulfide minerals with water, ferric iron and oxygen [79].

**Figure 2:**
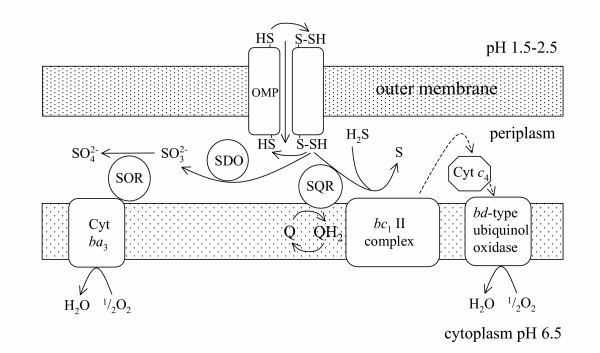
A composite model of sulfur oxidation electron transport pathway of *At. ferrooxidans *based on references [10, 76, 96]. Thiol groups of outer membrane proteins are believed to transport the sulfur to the periplasm where it is oxidized by a periplasmic sulfur dioxygenase (SDO) to sulfite and a sulfite acceptor oxidoreductase (SOR) to sulfate [76]. Although other cytochrome oxidases are present, a *ba*_3 _cytochrome oxidase and a *bc*_1 _II complex together with a *bd*-type ubiquinol oxidase are believed to play the major roles during sulfur oxidation [10, 96]. Rusticyanin and an iron oxidizing protein (not shown) might also be involved during sulfur oxidation but their exact role is still to be determined [96].

Attempts to investigate the pathways involved in sulfur oxidation by acidophilic bacteria have proved challenging. The chemical reactivity of many sulfur intermediates has meant that some intermediates may be produced by a combination of spontaneous and enzymatic reactions [76,79]. Nevertheless, progress has being made. Working with *At. ferrooxidans*, *At thiooxidans *and the RISC-oxidizing *Acidiphilium acidophilium*, Rohwerer and Sand [76] proposed a model for the oxidation of elemental and free sulfide sulfur. Extracellular elemental sulfur is mobilized by the thiol groups of specific outer membrane proteins and transported into the cytoplasm as persulfide sulfane sulfur (see Figure 2). This persulfide sulfur is oxidized further to sulfate by a sulfite:acceptor oxidoreductase with the electrons most likely being transferred to cytochromes. Glutathione plays a catalytic role in elemental sulfur activation but is not consumed during enzymic sulfane sulfur oxidation. Sulfide oxidation required the disulfide of glutathione which reacted non-enzymatically with sulfide to give glutathione persulfide prior to enzymic oxidation. Free sulfide is oxidized to elemental sulfur in the periplasm by a separate sulfide:quinone oxidoreductase. Reaction with the thiol groups of the outer membrane proteins keeps the zero valence sulfur from precipitating in the periplasm.

In a study of the proteins induced when *At. ferrooxidans *cells were grown on sulfur compared with iron, it was found that an outer membrane protein, a putative thiosulfate sulfur transfer protein, a putative thiosulfate/sulfate binding protein, a putative capsule polysaccharide export protein and several other proteins of unknown function were induced [67]. The thiosulfate sulfur transfer protein and the thiosulfate/sulfate binding proteins appeared to be transcriptionally linked to a gene for a terminal oxidase. Several other proteins involved in sulfur oxidation have also been identified including a sulfur dioxygenase, a rhodanase and a 40 kD outermembrane protein. However, which proteins are required for the oxidation of different RISCs is far from being understood. Furthermore, studies on the biochemistry of sulfur oxidation including evidence for a *bc*_1 _complex and several cytochrome oxidases (*bd *and *ba*_3_) that are produced in higher concentrations when grown on sulfur than iron have been reported [10]. A model in which the components of iron and sulfur oxidation both feed electrons into an *aa*_3_-type cytochrome *c *oxidase has been proposed to account for biochemical and gene expression data [96]. There are indications that there may be more uniformity in the pathways used by at least the Gram-negative sulfur-oxidizing bacteria [30,76] than there is in iron oxidation pathways. This probably does not stretch to the sulfur-oxidizing archaea where thiol independent systems have been isolated. Irrespective of the pathway used, the ultimate oxidation product of RISCs is sulfate and this results in a decrease in pH.

#### 7.3 Other sources of energy

Soluble metal ions are frequently present fairly high concentrations in highly acidic environments. Metal ions which exist in more than one oxidation state and which have redox potentials that are more negative than the O_2_/H_2_O redox couple, have the potential to serve as electron donors for acidophilic bacteria. An *At. ferrooxidans-*like bacterium was reported to directly oxidize Cu^+ ^to Cu^2+ ^[51,53] and U^4+ ^to U^6+ ^under aerobic conditions and that these oxidation reactions were coupled to CO_2 _fixation [24]. However, whenever ferric iron is present, it is difficult to unequivocally demonstrate the biological oxidation of the metal as opposed to chemical oxidation of the metal by ferric iron. Similarly it has been reported that Mo^5+ ^can be oxidized to Mo^6+ ^and a molybdenum oxidase has been isolated from cell extracts of *At. ferrooxidans *[85]. The potential also exists that the oxidation of oxyanions such as As^3+ ^(AsO_2_^-^) to As^5+ ^(AsO_4_^3-^) can serve as an alternate electron donor for acidophilic organisms [83]. An analysis of the *At. ferrooxidans *ATCC23270 genome revealed that as many as eleven cytochromes *c *were present [98]. One cytochrome *c *was specific for growth on sulfur, three were specific for growth on iron and several were produced on both substrates. The large number of cytochrome *c *molecules might also be a reflection of the versatility of electron donors (and electron acceptors) that the bacterium is capable of using.

The type strain of *At. ferrooxidans *ATCC23270 as well as the two other *At. ferrroxidans *strains tested were found to grow by hydrogen oxidation but not *At. thiooxidans *or *L. ferrooxidans *[27]. When growing on hydrogen they had a broad pH optimum of pH 3.0 to 5.8 with no growth occurring at pH<2.2 or pH>6.5. Hydrogen oxidation appeared to be repressed by the presence of S^0^, Fe^2+ ^and sulfidic ore. In a later study, only one of six *At. ferrooxidans *strains tested could use hydrogen as an electron donor to support CO_2 _fixation and cell growth with oxygen as electron acceptor [58]. There is a possibility that some isolates of the genes *Leptospirillum *might be able to use hydrogen as an electron donor although this has not yet been demonstrated.

### 8. Relationship to oxygen and alternate electron acceptors

The chemolithotrophic acidophiles require large quantities of energy to support their autotrophic lifestyle. As may be expected, their most commonly used terminal electron acceptor is oxygen as this is energetically the most favourable option. As described earlier, the redox potential of the Fe^2+^/Fe^3+ ^couple is almost as positive as that of O_2_/H_2_O and consequently ferric iron is a potentially suitable alternate electron acceptor. For an autotrophic acidophile to be able to use ferric iron as electron acceptor it must be capable of using RISCs or molecules other than ferrous iron as an electron donor. The oxidation of sulfur and tetrathionate coupled to ferric iron reduction under anaerobic conditions has been shown to occur in the case of *At. ferrooxidans *[88]. It has also been shown that several though not all isolates of this bacterium can grow by using the H_2_- or S^0^-coupled reduction of ferric iron [58]. Other autotrophic sulfur-oxidizers like *At. thiooxidans *and *At. caldus *are apparently unable to catalyze the reduction of ferric iron in the absence of air [35]. Besides the ability to use ferric iron, the *At. ferrooxidans *is also able to reduce Mo^6+^, Cu^2+ ^and Co^2+ ^when using elemental sulfur as an electron donor [86,87]. *At. ferrooxidans *and *At. thiooxidans *have been reported to reduce V^5+ ^to V^4+^, however, whether the oxidized vanadium served as an electron acceptor for respiration was unclear as the shake flasks were aerated [11]. As described earlier, the large variety of cytochrome *c *molecules might reflect the versatility of *At. ferrooxidans *to use a wide variety of electron acceptor.

The potential to grow by ferric iron respiration is even greater amongst the extremely acidophilic heterotrophs since ferric iron reduction can be coupled to the oxidation of many organic compounds. Indeed some *Acidiphilium *species are able to reduce ferric iron even under aerobic conditions such as in shake flasks and on the surface of agar plates, although ferric iron reduction is enhanced when the oxygen concentrations are relatively low [44]. Furthermore, not only soluble but also insoluble amorphous or crystalline minerals such Fe(OH)_3 _and jarosite can be reductively solubilized by *Acidiphilium *SJH using ferric iron [12]. Ferric iron respiration has the advantage of regenerating additional ferrous iron electron donor for the iron-oxidizing obligate autotrophs should aerobic conditions again prevail.

### 9. Acidophilic properties

From an industrial perspective it is essential that biomining microorganisms are able to grow at low pH and tolerate high concentrations of acid. Two important reasons for this are to enable iron cycling and to permit reverse electron transport to take place.

A low pH is required for the iron cycle whereby ferrous iron serves as an electron donor under aerobic conditions and ferric iron as an energetically favourable alternate electron acceptor if the concentration of oxygen falls. This has been described above. Ferric iron is almost insoluble at a neutral pH, whereas in acid solutions its solubility is increased. The possibility of using ferric iron as an alternate electron acceptor is therefore readily available to acidophiles but less available to aerobic neutrophiles or moderate acidophiles because ferric iron is almost totally insoluble in neutral, aerobic environments.

The external pH of the environment in which extreme acidophiles such as biomining microbes grow is low (e.g. pH 1.0–2.0), whereas the internal cellular pH remains close to neutral [20]. This difference results in a steep pH gradient across the cell membrane. This pH gradient is important for nutritional purposes, especially when using a weak reductant such as ferrous iron as an electron donor. Autotrophic organisms have a high requirement for compounds such as NAD(P)H to reduce their carbon source (CO_2_) to produce the sugars, nucleotides, amino acids and other molecules from which new cell mass is synthesized. Heterotrophic bacteria do not have as high a demand for NAD(P)H as their carbon source is more reduced than CO_2 _and hydrogen atoms removed from their source of nutrition may be used to satisfy their lower NAD(P)H requirement. Chemolithotrophic autotrophs require a large transmembrane proton gradient to generate the required proton motive force to energise the synthesis of NAD(P)H. This process is known as reverse electron transport or the 'uphill' electron transfer pathway [9]. Although this phenomenon has not been studied in many iron- or sulfur-oxidizing chemolithotrophs, strong evidence has been presented that when grown on iron, *At. ferrooxdians *contains a unique cytochrome *bc*_1 _complex that functions differently from the *bc*_1 _complex used during the oxidation of sulfur and is specifically involved in the 'uphill' pathway [28]. One way of viewing this is that growth in acid solutions is a nutritional necessity as a large transmembrane pH gradient is required to produce the hydrogen atoms needed to reduce CO_2 _to cell mass.

### 10. Adaptability and ability to compete in a non-sterile environment

In many industrial processes that are dependent on the use of microorganisms it is important that the process is kept largely free from contamination by undesired organisms. From the description of biomining processes given in the introduction it is clear that 'non-sterile' open stirred tanks or heaps exposed to the environment are used. Such processes are susceptible to 'contamination' by microorganisms present on the ores, concentrates, inorganic nutrient solutions, water air etc. Given the huge volumes of mineral that have to be processed, the relatively low value of the product and nature of a mining environment the cost-effective prevention of contamination would be impossible to achieve. Fortunately this is not required. The aim of the process is the biodegradation of the mineral or concentrate and one seeks organisms that are able to do this most effectively. Those microorganisms that are able to degrade the mineral most effectively are also those that grow the quickest and therefore have the fastest doubling times. In a continuous-flow process such as provided by a series of completely mixed leaching tanks, microorganisms in the tanks are continually being washed out. There is thus a strong positive selection for microbes that grow most effectively on the mineral as those microbes that grow and divide the fastest are subjected to less wash out and will dominate the microbial population in the biooxidation tanks. There are few biological fermentation processes that share this advantage with another notable example being activated sludge sewage treatment process where organisms with the capacity to grow most effectively on the waste in the water are selected.

Previous unreported research experience by the author has found that after a period of operation, the metabolic capabilities of a population of biomining organisms may improve out of all recognition from the culture originally inoculated into the tanks. One would predict that natural populations of microorganisms are adapted for survival under the highly variable feast or famine conditions that are experienced in nature rather than the optimized, controlled conditions of a biooxidation tank. Early experiments on gold-biooxidation were carried out in a series of three or four continuous-flow, aerated, stirred tank reactors. As these reactors are expensive to construct and operate, the rate of concentrate decomposition has an important effect on the economics of the process [23]. The initial process was very slow because unadapted cultures of biooxidation bacteria were probably not tuned to rapid growth and possibly also because they were sensitive to the arsenic released from the arsenopyrite. Initially a retention time of over twelve days was required for sufficient biooxidation to allow more than 95% gold recovery [73]. However, a period of selection of about two years in a laboratory scale and then pilot plant scale continuous flow process resulted in a reduction in the retention time of concentrate in the reactors to seven days. During the first two years of operation in a full-scale continuous-flow biooxidation plant the growth rate of the bacteria had improved still further so that the retention time had been reduced to about 3.5 days. At the same time the solid concentration in the liquor was increased from 10 to 18% so that the same equipment could be used to treat almost four times the amount of concentrate per day as initially. This process was developed by Gencor SA [23,93] and registered as the Biox process.

### 11. Metal tolerance and resistance

An important characteristic of the acidophilic chemolithotrophs is their general tolerance of high concentrations of metallic and other ions. The levels of resistance of several acidophilic bacteria and archaea to As^3+^, Cu^2+^, Zn^2+^, Cd^2+ ^and Ni^+ ^have recently been reviewed and will not be covered here in detail [25]. As may be predicted, levels of resistance show considerable strain variation. Adaptation to high levels of metal resistance on exposure to a metal is likely to be responsible for much of the variation. *At. ferrooxidans *appears to be particularly resistant to metals and the bacterium has been reported to grow in medium containing Co^2+ ^(30 g/l), Cu^2+ ^(55 g/l), Ni^2+ ^(72 g/l), Zn^2+ ^(120 g/l), U_3_O_8 _(12 g/l) and Fe^2+ ^(160 g/l). In a comparative study of two *At. ferrooxidans*, two *L. ferrooxidans *and an *At. thiooxidans *strain, it was found that *At. ferrooxidans *and *L. ferrooxidans *were approximately equally resistant to Cu^2+^, Zn^2+^, Al^3+^, Ni^2+ ^and Mn^2+^, but that *L. ferrooxidans *was more sensitive (<2 g/l) than *At. ferrooxidans *to Co^2+ ^[77]. *At. thiooxidans *was sensitive to less than 5 g/l of all the cations used in the comparative study with the exception of Zn^2+ ^(10 g/l). No studies have been carried out on the molecular mechanisms of metal resistance in any of these bacteria.

Genome sequencing data on *At. ferrooxidans *and *Leptospirillum *type II plus work from many other groups suggest that metal resistance is due to a combination of genes that are probably present on the chromosomes of most isolates of a bacterial species and mobile genes acquired by specific isolates of a species. An example of genes present on the chromosomes of most species of a genes are the efflux genes for arsenic [15], copper, silver cadmium and several metal cations in *At. ferrooxidans *(genome sequence data, [3]). Another example of a resistance mechanism that might be present in all members of a species because it is associated with general cell physiology is the polyphosphate mechanism for copper resistance of *At. ferrooxidans *[1]. These workers presented a model whereby the hydrolysis of polyphophates resulted in the formation of metal-phosphate complexes that are transported out of the cell enhancing resistance to the metal.

Mobile genes for metal or metalloid resistance that might be present in certain isolates but not others of the same species are genes present on plasmids or transposons. These genes may be recruited from the horizontal gene pool by the acquisition of a plasmid or the insertion of metal resistance containing transposons into either the chromosome or a plasmid. For example, when ten *At. ferrooxidans *isolates were screened for Hg^+ ^resistance, three of the strains contained DNA that hybridized to a Tn*501 mer *gene probe [82]. Bacteria carrying the resistance genes were in general 3–5 times more resistant to Hg^2+ ^than strains that did not have *mer *genes. The *mer *genes of the E-15 strain of *At. ferrooxidans *were cloned and sequenced and truncated transposon Tn*7*-like fragments were found in the vicinity [39,40]. Codon usage analysis suggested that the *mer *genes had originated from an organism different from *At. ferrooxidans *[40]. A Tn*21*-like transposon (Tn*5037*) that contains mercury resistance genes was isolated from another strain of *At. ferrooxidans *G66 [46]. Some strains of *At. ferrooxidans *appear to contain a mercury resistance mechanism that is so far unique to the species. Mercury volatilization in these strains was dependent on Fe^2+ ^as an electron donor but not NADPH as found with other mercury resistance mechanisms [42]. The cytochrome *c *oxidase appeared to deliver electrons directly to mercury (Figure 1) [84]. It was possible to take *At ferrooxidans *SUG2.2 cells already resistant to 6 μM Hg^2+ ^and adapt them by successive cultivation to produce *At. ferrooxidans *strain MON-1 that was resistant to 20 μM Hg^2+^. This property was maintained after several rounds of cultivation on iron in the absence of Hg^2+^. Interestingly, rusticyanin from mercury resistant cells enhanced Fe^2+^-oxidation actitity of plasma membranes and activated Fe^2+^-dependent mercury volatilization activity [42]. This supports the view of Rohwerder et al. [75] that rusticyanin serves as a channel of electrons from iron. Comparison of cytochrome *c *oxidases from *At. ferrooidans *strains that are resistant to Hg^2+^, Mo^5+^, sulfite and 2,4 dinitrophenol with sensitive strains led the authors to suggest that different cytochrome *c *oxidases.might be responsible for resistance to different substances by related mechanism [84].

An example of where resistance genes may be acquired from the horizontal gene pool when needed are the arsenic resistance genes recruited by *At. caldus *[21,90] and *L. ferriphilum *(unpublished). These two bacteria have been shown to dominate the biooxidation tanks used to treat gold-bearing arsenopyrite concentrate at the Fairview mine [71]. When microorganisms capable of rapidly oxidizing arsenopyrite concentrate in continuous flow aeration tank were being selected, the rates of oxidation were initially slow. One of the reasons for this is that the organisms were sensitive to arsenic. Once arsenic levels had built up in solution above 1 g/l total arsenic, the process slowed and arsenic had to be precipitated and removed from solution by raising the pH. After arsenic removal and subsequent aeration, biooxidation rates increased until the concentration of arsenic in solution again built up and the arsenic was reprecipitated. After almost two years of selection in continuous-flow laboratory and pilot scale tanks, the microorganisms had become sufficiently resistant to the 13 g/l total arsenic in solution for arsenopyrite biooxidation to take place without the need to remove the arsenic. Unfortunately, the original unadapted arsenic sensitive culture was not maintained and therefore was not available to compare with the highly arsenic resistant culture present in the commercial Biox^® ^plant at the Fairview mine in 1996 when arsenic resistance mechanisms were investigated (approximately ten years after it had been commissioned). *L. ferriphilum *and *At. caldus *strains were isolated from the Biox^® ^tanks and their arsenic resistance mechanisms examined and compared with those of the same species of bacterium that were not known to have been previously exposed to arsenic.

**Figure 3:**
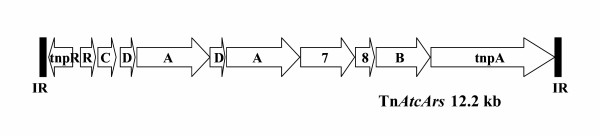
The arsenic resistance gene containing transposon, Tn*AtcArs*, present in highly arsenic resistant strains of *At. caldus *[90]. The arsenic resistance genes are located between the inverted repeat sequences (IR), resolvase (tnpR) and transposase (*tnpA*) genes of the Tn*21*-like transposon. R, arsenic resistance regulator; C, arsenate reductase; D, upper-limit arsenic regulator; A, arsenite efflux-dependent ATPase; 7, ORF with a NADH oxidoreductase domain; 8, ORF with a CBS-like domain; B, membrane arsenite efflux transporter.

Studies on arsenic resistance genes of six strains of isolates of *At. caldus *were carried out, three with known exposure to arsenic and three without. Of the three strains previously exposed to arsenic, one strain originated from the Biox^® ^plant at Fairview, another from a pilot plant oxidixing arsenopyrite at the University of Cape Town and third from a culture used to treat a nickel-containing ore but which was derived from same culture used in the Fairview plant. Of the three *At. caldus *isolates not known to have been exposed to arsenic, one originated from Australia and two from the United Kingdom. DNA-DNA hybridization experiments indicated that all six strains contained a set of arsenic resistance genes present on their chromosomes. However, the three arsenic resistant strains contained arsenic resistance genes in addition to those present in all strains. The arsenic resistance genes were present on a transposon belonging to the Tn*21 *family that must have been acquired from the horizontal gene pool. All three resistant strains contained a copy of the Tn*AtcArs *transposon (Figure 3) and at least one strain had an additional incomplete copy of the transposon [90]. The arsenic resistance genes were arranged in an unusual manner with the *arsA *(ATPase) and *arsD *(regulator and provision of arsenite) being duplicated. In the *At. caldus *strain isolated from the nickel plant, the *arsA *and *arsD *duplication was absent. Efforts are being made to introduce Tn*AtcArs *into arsenic sensitive strains of *At. caldus *to determine the contribution of Tn*AtcArs *to arsenic resistance of the host. A question to be addressed, is from where did the Tn*AtcArs *acquired by the arsenic resistant strains originate? DNA sequencing data indicated that the closest relative to the *ars *gens is on a transposon present in a heterotrophic bacterium *Alcaligenes faecalis*. The percentage amino acid sequence identity of proteins associated with arsenic resistance on the two transposons was high (70–95%) but not identical. This suggests that the two transposons originated from the same ancestral plasmid. However, the differences are sufficient to suggest that the two transposons have evolved independently for many years (difficult to allocate a time scale) and that *At. caldus *and *A. faecalis *did not originate from the same gene pool at the time that the arsenic resistant *At. caldus *strains were exposed to high levels of arsenic in the early 1980's.

The account of arsenic resistance gene acquisition just described is an illustration of an advantage to be gained by the bioleaching and biooxidation processes being non-sterile, open systems. New organisms will continually enter the system and the iron- and sulfur-oxidizing microbes present will have the opportunity of accessing the horizontal gene pool that these organisms contain and that are selected by growth conditions.

### 12. Conclusion

The solubilization of metals from minerals or their concentrates is believed to be largely a chemical process that is due to the action of ferric iron and protons depending on the mineral being treated. Like all chemical processes, the rate of reaction is affected by temperature. Some difficult-to-degrade minerals need to be leached at higher temperatures than others for the leaching reactions to proceed at an economically viable rate. Since microorganisms are responsible for producing the leaching reagents and because contact between the microbes and the mineral speeds up the process, there is a need for microorganisms to be able to produce the leaching reagents at a variety of temperatures.

As would be expected, the types of microorganisms present in processes used for the recovery of metals vary hugely depending on the temperature at which the process is carried out. Commercial processes that operate at temperatures from ambient to 40°C are dominated by Gram-negative bacteria with some *Ferroplasma*-like organisms being present if the pH drops below about pH 1.3. There is some overlap with bacteria that dominate processes that operate at 40°C with those at 45–55°C (e.g. *L. ferriphilum *and *At. caldus*), but there are also some clear differences. In particular Gram-positive bacteria belonging to the genus *Sulfobacillus *appear to play a significant role at the higher temperatures and archaea of the *Ferroplasma *type are more frequently found. In contrast, microorganisms present in processes that operate at 75–80°C are all archaea. Although there are no commercial processes currently operating in the range 60–70°C suitable organisms almost certainly exist and are likely to be present in low pH hot sulfur springs. The variation in microorganism present in a bioleaching process appears to be more dependent on temperature than on the type of iron-and sulfur-containing mineral being oxidized or on whether tank or heap reactors are being used.

In spite of the large variety of potential organisms that can be used, the microbes that play the most important roles tend to have certain properties in common. They obtain their energy by the oxidation of either iron or reduced inorganic sulfur compounds. Although some microorganisms are capable of using both energy sources, a combination of iron-oxidizing and sulfur-oxidizing microbes often works best. The production of sulfuric acid and the need to keep the most important mineral-oxidizing agent (ferric iron) in solution means that the organisms are acid tolerant. The iron- and sulfur-oxidizing organisms are, in general, autotrophic and do not require to be provided with an external carbon source. When in pure culture, some grow better with small amounts of yeast extract or if aerated with CO_2_-enriched air. However, when growing in a mixed microbial consortium, cross-feeding appears to take place so that an extra source of carbon is not required. The microorganisms tend to be resistant to high concentrations of metal ions and where this is lacking they have demonstrated a remarkable ability to become metal-resistant. At least some of this metal resistance is due to the acquisition of metal genes from the horizontal gene pool.

*At. ferrooxidans *is the first bacterium that was recognized as being present in bioleaching environments. This bacterium has been more extensively studied than any other biomining organism and was also the first to have its genome sequenced [3]. Although this bacterium is readily isolated from acid mine drainage and heap reactors operating below 40°C, it appears not to be the most important leaching organism in most high-rate commercial processes. In depth studies on several of the other types of biomining organisms is therefore also needed. The recent gapped genome sequences of *L. ferriphilum *and a strain of *Ferroplasma *were assembled during an environmental metagenome project on the organisms present in acid mine drainage [92]. This and other genome sequencing projects being planned should provide assistance in expanding our knowledge on other important biomining microbes.
